# Comparison between drug-eluting bead transarterial chemoembolization versus conventional TACE in elderly patients with colorectal cancer liver metastases: a cohort study

**DOI:** 10.3389/fonc.2026.1785622

**Published:** 2026-06-10

**Authors:** Liying Tian, Bao Hu, Meifang Song, Yunpeng Li, Fan Yang, Jiecheng Yang

**Affiliations:** 1 The Second Ward of the Department of Gastroenterology, Handan Central Hospital, Handan, China; 2 The Second Department of Vascular Intervention, Handan Central Hospital, Handan, China; 3 Emergency Surgery, Beijing Children’s Hospital Capital Medical University, Beijing, China; 4 Cardiovascular Surgery, Hebei Medical University Third Hospital, Shijiazhuang, China; 5 Child Care, Merice Cody Public School, Toronto, ON, Canada

**Keywords:** colorectal cancer liver metastases, conventional TACE, DEB-TACE, efficacy, safety

## Abstract

**Background:**

Drug-eluting bead (DEB) transarterial chemoembolization (TACE) possesses several advantages over conventional TACE (cTACE) such as higher intratumor drug concentration and lower systemic toxicity, which has been widely applied in hepatocellular carcinoma patients, but related information is insufficient in patients with colorectal cancer liver metastases (CRCLM). This study aimed to investigate the efficacy and safety of DEB-TACE versus cTACE in treating elderly CRCLM patients.

**Methods:**

A total of 43 elderly patients with unresectable CRCLM were retrospectively analyzed in this cohort study, among which 20 cases received DEB-TACE and 23 cases received cTACE. Best treatment response, progression-free survival (PFS), overall survival (OS), and adverse events (AEs) were collected.

**Results:**

Baseline characteristics were comparable between the DEB-TACE and cTACE groups, with no statistically significant differences observed. Complete response rate (10.0% versus 4.3%, *P* = 0.468), objective response rate (55.0% versus 30.4%, *P* = 0.103), and disease control rate (95.0% versus 82.6%, *P* = 0.206) showed higher tendencies in the DEB-TACE group compared with the cTACE group but did not reach statistical significance. PFS was longer in the DEB-TACE group compared with the cTACE group [median: 11.7 (95% CI: 7.5–15.9) versus 6.1 (95% CI: 5.1–7.1) months, *P* = 0.013], but OS only exhibited a prolonged tendency without statistical significance [21.6 (95% CI: 14.3–28.9) versus 16.3 (95% CI: 11.6–21.0) months, *P* = 0.076]. The incidence of AEs was not different between the DEB-TACE and cTACE groups, including fever, pain, nausea and vomiting, ALT elevation, AST elevation, anemia, leukopenia, and thrombocytopenia.

**Conclusion:**

DEB-TACE can be an optional choice for the treatment of elderly CRCLM patients.

## 1 Introduction

Colorectal cancer (CRC) is the second leading cause of cancer-related deaths, accounting for 9.3% of all sites (1). The optimal option for CRC treatment is radical resection with or without perioperative therapies (2, 3); however, approximately 15%–30% of CRC has distant metastases, among which the liver is the most common metastatic site, worsening life expectancy (4). For colorectal cancer liver metastases (CRCLM), some patients can benefit from liver metastasis resection (5), but in other patients who are unsuitable or unwilling to receive resection, they can only receive non-curative standard chemotherapy, targeted therapy, immunotherapy, and regional therapy (6, 7).

Drug-eluting bead transarterial chemoembolization (DEB-TACE) is a latest regional therapeutic technology that simultaneously achieves tumor embolization and targeted chemotherapy (8, 9). DEB-TACE utilizes microspheres as drug carrier and embolism material for the transarterial chemoembolization (TACE) procedure instead of lipiodol and gelatin sponge as that in conventional transarterial chemoembolization (cTACE), which can achieve a higher intratumor chemotherapeutic drug concentration and less systemic toxicity (10, 11). DEB-TACE has been widely used in hepatocellular carcinoma (HCC) patients, exhibiting a satisfied efficacy and acceptable tolerance (12–14). Moreover, a few studies preliminarily explored the application of DEB-TACE in CRCLM patients, but these studies were mainly single arm or had limited sample sizes (15–17), and investigations involving elderly CRCLM patients are lacking.

Elderly patients with CRCLM typically present with fewer liver lesions, receive less perioperative chemotherapy, and undergo less extensive resections (18). Moreover, postoperative outcomes show increased mortality with advancing age, presenting a pooled risk ratio of 2.53 for postoperative mortality in older versus younger patients; therefore, resection may not be the preferred initial treatment option for this population (19). In such conditions, TACE provides a choice for elderly patients with CRCLM (20, 21). However, the advantages and disadvantages between DEB-TACE and cTACE in those patients are seldom explored previously.

This present study aimed to investigate the efficacy and safety of DEB-TACE compared with cTACE in treating elderly patients with CRCLM.

## 2 Materials and methods

### 2.1 Participants

A total of 43 elderly patients with unresectable CRCLM between October 2020 and November 2023 in Handan Central Hospital and Hebei Medical University Third Hospital were analyzed in this multicenter, retrospective, cohort study. Among them, 37 patients were from Handan Central Hospital and 6 patients were from Hebei Medical University Third Hospital. The key inclusion criteria included the following: 1) primary CRC diagnosis by pathological confirmation; 2) primary CRC that has been resected; 3) liver metastases confirmed by pathological test; 4) liver metastases deemed unsuitable for resection or patients unwilling to receive resection; 5) receipt of DEB-TACE or cTACE for liver metastases; 6) age ≥60 years; 7) Eastern Cooperative Oncology Group (ECOG) performance score of 0–2; and 8) at least one measurable lesion that could be assessed via the modified Response Evaluation Criteria in Solid Tumors (mRECIST) criteria. The key exclusion criteria included 1) other metastases apart from liver and 2) without any follow-up data for evaluation. Elderly was defined as ≥60 years old in this study, which referred to the “Law of the People’s Republic of China on the Protection of the Rights and Interests of the Elderly”; however, this was a regional administrative definition. This study was approved by the Ethics Committee of Handan Central Hospital with the approval number HDSZXYY2023120008. This study was performed adhering to the Declaration of Helsinki. The patients or their families provided informed consent for data use.

### 2.2 Data collection

Baseline data were collected from CRCLM patients including age, sex, ECOG performance score, Child–Pugh stage, time to liver metastases, location of liver metastases, number of liver metastases, largest diameter of liver metastases, primary tumor site, Kirsten rat sarcoma viral oncogene homolog (KRAS) mutation, v-Raf murine sarcoma viral oncogene homolog B (BRAF) V600E mutation, and lines of prior systemic therapies. The data were retrieved from the electronic medical system.

### 2.3 The TACE procedure

DEB-TACE was performed using microspheres as the drug carrier and embolism material for the procedure, while cTACE was performed using lipiodol and gelatin sponge as the drug carrier and embolism material for the procedure. Irinotecan (Jiangsu Hengrui Pharmaceuticals, China) was applied as a chemotherapeutic drug for TACE. Microspheres included DC beads^®^ (100–300 μm, Boston Scientific, USA) and CalliSpheres^®^ (300–500 μm, Suzhou Hengrui Jiali Sheng Biomedical Technology, China). The actual median (range) volume of DC beads^®^ was 1.0 (0.8–1.7) vial, and the actual median (range) volume of CalliSpheres^®^ was 1.0 (0.9–1.8) vial. Each vial of microspheres was mixed with 100 mg of irinotecan, and the actual median (range) dose of irinotecan was 100 (80–180) mg in the DEB-TACE group.

TACE was performed referring to the universal standard guidelines. In brief, the distribution of tumor vessels was determined by digital subtraction angiography, and femoral artery puncture was performed using the Seldinger technique. Then, the hepatic artery catheter and microcatheter (2.7Fr, Terumo, Japan) were inserted into the feeding artery of the liver metastases. In the DEB-TACE procedure, the chemotherapy drug was mixed with microspheres and injected into the feeding artery. In the cTACE procedure, the chemotherapy drug was mixed with lipiodol and injected into the feeding artery, with gelatin sponge particles added. The embolization endpoint was the disappearance of tumor staining. In general, the average TACE times (treatment cycle) were 1.6 times in the DEB-TACE group and 1.9 times in the cTACE group.

### 2.4 Outcomes

Imaging examination was performed via computed tomography or magnetic resonance imaging, at approximately 1 month after the first procedure of TACE and then every 2–3 months. According to the imaging examination, treatment response was evaluated on the basis of mRECIST, and the best treatment response was retrieved for analysis. According to the follow-up data, progression-free survival (PFS) and overall survival (OS) were analyzed. Moreover, the occurrences of adverse events including fever, pain, nausea and vomiting, alanine aminotransferase (ALT) elevation, aspartate aminotransferase (AST) elevation, anemia, leukopenia, and thrombocytopenia were also retrieved and analyzed. ALT elevation indicated ALT above the upper limit of normal (ULN), AST elevation indicated AST above the ULN, anemia indicated hemoglobin below the lower limit of normal (LLN), leukopenia indicated white blood cell count below the LLN, and thrombocytopenia indicated platelet count below the LLN.

### 2.5 Statistical analysis

The data are shown as mean ± standard deviation (SD) or number (percentage). Specifically, age is shown as mean ± SD; sex, ECOG performance score, Child–Pugh stage, and location of liver metastases are shown as number (percentage); the number of liver metastases, the largest diameter of liver metastases, and the time to liver metastases are categorized by the corresponding thresholds and shown as number (percentage). Comparisons of data between the two groups were performed using *t*-test or chi-square test as appropriate. PFS and OS between the two groups were presented by Kaplan–Meier curves and compared by the log-rank test. Variables related to PFS or OS were firstly screened by univariable Cox regression, and then variables with *P*-value <0.1 in the univariable Cox regression were further included in the multivariable Cox regression analysis. Statistical analysis was performed using SPSS Statistics software (IBM, USA), and a graph was drawn using GraphPad Prism software (Dotmatics, USA), respectively. A *P*-value <0.05 was considered statistically significant.

## 3 Results

### 3.1 Comparison of baseline characteristics

The age was 70.9 ± 6.6 years in the DEB-TACE group and 68.8 ± 6.1 years in the cTACE group (*P* = 0.291). The male percentage was 80.0% in the DEB-TACE group and 69.6% in the cTACE group (*P* = 0.434). ECOG performance scores of 0, 1, and 2 were observed in 25.0%, 65.0%, and 10.0% of patients from the DEB-TACE group and in 8.7%, 73.9%, and 17.4% of patients from the DEB-TACE group (*P* = 0.163). Moreover, 10.0%, 55.0%, 40.0%, 60.0%, and 40.0% of patients had Child–Pugh stage B, time to liver metastases <1 year, bilobar disease, multifocal disease, and largest diameter of liver metastases ≥5 cm in the DEB-TACE group, while the corresponding proportions were 13.0%, 65.2%, 47.8%, 69.6%, and 52.2% in the cTACE group (statistically non-significant). In addition, primary tumor location, KRAS mutation, BRAF V600E mutation, and lines of prior systemic therapies were not different between the DEB-TACE group and the cTACE group (**Table 1**).

**Table 1:** Characteristics of CRCLM patients.

Items	cTACE group (*N* = 23)	DEB-TACE group (*N* = 20)	*P*-value
Age (years), mean ± SD	68.8 ± 6.1	70.9 ± 6.6	0.291
Sex, *n* (%)			0.434
Female	7 (30.4)	4 (20.0)	
Male	16 (69.6)	16 (80.0)	
ECOG performance score, *n* (%)			0.163
0	2 (8.7)	5 (25.0)	
1	17 (73.9)	13 (65.0)	
2	4 (17.4)	2 (10.0)	
Child–Pugh stage, *n* (%)			0.756
Stage A	20 (87.0)	18 (90.0)	
Stage B	3 (13.0)	2 (10.0)	
Time to liver metastases, *n* (%)			0.494
≥1 year	8 (34.8)	9 (45.0)	
year	15 (65.2)	11 (55.0)	
Location of liver metastases, *n* (%)			0.606
Unilobar	12 (52.2)	12 (60.0)	
Bilobar	11 (47.8)	8 (40.0)	
Number of liver metastases, *n* (%)			0.512
Unifocal	7 (30.4)	8 (40.0)	
Multifocal	16 (69.6)	12 (60.0)	
Largest diameter of liver metastases, *n* (%)			0.425
cm	11 (47.8)	12 (60.0)	
≥5 cm	12 (52.2)	8 (40.0)	
Primary tumor site, *n* (%)			0.517
Right colon	13 (56.5)	8 (40.0)	
Left colon	5 (21.7)	7 (35.0)	
Rectum	5 (21.7)	5 (25.0)	
KRAS mutation, *n* (%)	8 (34.8)	8 (40.0)	0.724
BRAF V600E mutation, *n* (%)	2 (8.7)	1 (5.0)	1.000
Lines of prior systemic therapies, *n* (%)			0.420
1 line	4 (17.4)	3 (15.0)	
2 lines	10 (43.5)	6 (30.0)	
≥3 lines	9 (39.1)	11 (55.0)	

CRCLM, colorectal cancer liver metastases; cTACE, conventional transarterial chemoembolization; DEB-TACE, drug-eluting bead transarterial chemoembolization; SD, standard deviation; ECOG, Eastern Cooperative Oncology Group; KRAS, Kirsten rat sarcoma viral oncogene homolog; BRAF, v-Raf murine sarcoma viral oncogene homolog B.

### 3.2 Comparison of treatment response

The complete response (CR) rate, partial response (PR) rate, stable disease (SD) rate, and progressive disease (PD) rate were 10.0%, 45.0%, 40.0%, and 5.0% in the DEB-TACE group and 4.3%, 26.1%, 52.2%, and 17.4% in the cTACE group. By comparison, the treatment response was numerically better in the DEB-TACE group compared with the cTACE group, but it did not reach statistical significance (*P* = 0.077). Moreover, the CR rate (10.0% versus 4.3%, *P* = 0.468), objective response rate (ORR, 55.0% versus 30.4%, *P* = 0.103), and disease control rate (DCR, 95.0% versus 82.6%, *P* = 0.206) also showed higher tendencies in the DEB-TACE group compared with the cTACE group, but did not reach statistical significance (**Table 2**).

**Table 2:** Treatment response information.

Items	cTACE group (*N* = 23)	DEB-TACE group (*N* = 20)	*P*-value
Detailed treatment response, *n* (%)			0.077
CR	1 (4.3)	2 (10.0)	
PR	6 (26.1)	9 (45.0)	
SD	12 (52.2)	8 (40.0)	
PD	4 (17.4)	1 (5.0)	
CR rate, *n* (%)	1 (4.3)	2 (10.0)	0.468
ORR, *n* (%)	7 (30.4)	11 (55.0)	0.103
DCR, *n* (%)	19 (82.6)	19 (95.0)	0.206

cTACE, conventional transarterial chemoembolization; DEB-TACE, drug-eluting bead transarterial chemoembolization; CR, complete response; PR, partial response; SD, stable disease; PD, progressive disease; ORR, objective response rate; DCR, disease control rate.

### 3.3 Comparison of PFS and OS

**Figure 1:**
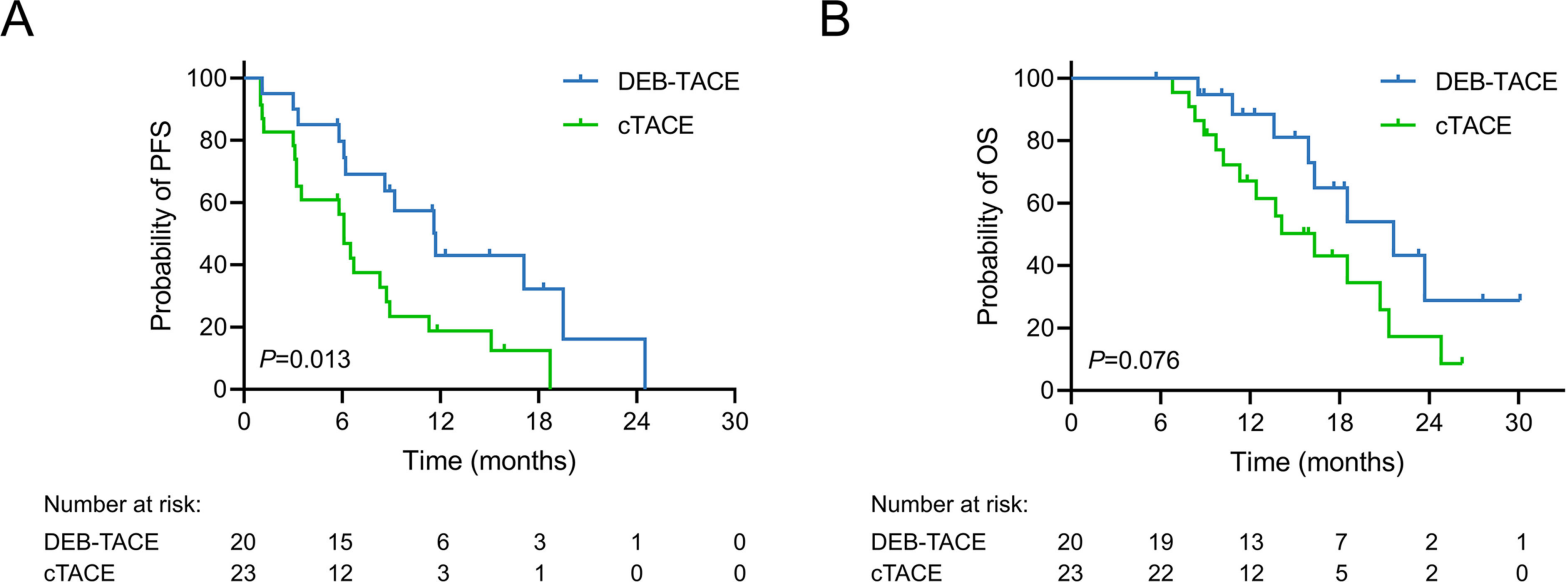
Kaplan–Meier curves. Comparison of PFS **(A)** and OS **(B)** between the DEB-TACE and cTACE treatments.

The median PFS was 11.7 (95% CI: 7.5–15.9) months in the DEB-TACE group, which was higher than that of 6.1 (95% CI: 5.1–7.1) months in the cTACE group (*P* = 0.013, **Figure 1A**). Moreover, the median OS was 21.6 (95% CI: 14.3–28.9) months in the DEB-TACE group, which showed a longer tendency compared to that of 16.3 (95% CI: 11.6–21.0) months in the cTACE group, but it did not reach statistical significance (*P* = 0.076, **Figure 1B**).

### 3.4 Factors related to PFS by Cox regression analyses

Univariable Cox regression analysis revealed that group (DEB-TACE versus cTACE) [hazard ratio (HR) = 0.405, *P* = 0.017] was correlated with longer PFS, but multifocal disease (HR = 2.182, *P* = 0.048), largest diameter of liver metastases ≥5 cm (HR = 2.290, *P* = 0.022), and higher lines of prior systemic therapies (HR = 1.752, *P* = 0.039) were correlated with shorter PFS (**Table 3**). Multivariable Cox regression analysis revealed that group (DEB-TACE versus cTACE) (HR = 0.291, *P* = 0.003) was independently correlated with better PFS, while higher lines of prior systemic therapies (HR = 2.206, *P* = 0.008) were independently correlated with worse PFS (**Table 4**).

**Table 3:** Univariable Cox regression for PFS.

Parameters	HR (95% CI)	*P*-value
Group (DEB-TACE versus cTACE)	0.405 (0.192–0.853)	0.017
Age ≥70 years	1.186 (0.584–2.406)	0.637
Sex—male	1.582 (0.698–3.587)	0.272
Higher ECOG performance score	1.667 (0.799–3.476)	0.173
Child–Pugh stage B	2.013 (0.765–5.300)	0.157
Time to liver metastases <1 year	1.504 (0.704–3.211)	0.292
Bilobar disease	1.756 (0.868–3.552)	0.117
Multifocal disease	2.182 (1.007–4.726)	0.048
Largest diameter of liver metastases ≥5 cm	2.290 (1.128–4.650)	0.022
Primary tumor site		
Reference (rectum)	1.000	
Right colon	1.311 (0.559–3.078)	0.534
Left colon	0.603 (0.208–1.749)	0.352
KRAS mutation	1.693 (0.807–3.555)	0.164
BRAF V600E mutation	3.276 (0.961–11.173)	0.058
Higher lines of prior systemic therapies	1.752 (1.029–2.982)	0.039

PFS, progression-free survival; HR, hazard ratio; CI, confidence interval; DEB-TACE, drug-eluting bead transarterial chemoembolization; cTACE, conventional transarterial chemoembolization; ECOG, Eastern Cooperative Oncology Group; KRAS, Kirsten rat sarcoma viral oncogene homolog; BRAF, v-Raf murine sarcoma viral oncogene homolog B.

**Table 4:** Multivariable Cox regression for PFS.

Parameters	HR (95% CI)	*P*-value
Group (DEB-TACE versus cTACE)	0.291 (0.128–0.665)	0.003
Multifocal disease	2.136 (0.899–5.079)	0.086
Largest diameter of liver metastases ≥5 cm	1.869 (0.862–4.055)	0.113
BRAF V600E mutation	3.013 (0.833–10.894)	0.093
Higher lines of prior systemic therapies	2.206 (1.226–3.968)	0.008

PFS, progression-free survival; HR, hazard ratio; CI, confidence interval; DEB-TACE, drug-eluting bead transarterial chemoembolization; cTACE, conventional transarterial chemoembolization; BRAF, v-Raf murine sarcoma viral oncogene homolog B.

### 3.5 Factors related to OS by Cox regression analyses

Univariable Cox regression analysis revealed that group (DEB-TACE versus cTACE) (HR = 0.468, *P* = 0.085) showed a tendency to be correlated with longer OS, but it did not reach statistical significance; otherwise, the largest diameter of liver metastases ≥5 cm (HR = 2.543, *P* = 0.031) and higher lines of prior systemic therapies (HR = 1.905, *P* = 0.044) were correlated with shorter OS (**Table 5**). Multivariable Cox regression analysis revealed that group (DEB-TACE versus cTACE) (HR = 0.382, *P* = 0.047) was independently correlated with better OS, but the largest diameter of liver metastases ≥5 cm (HR = 2.453, *P* = 0.046) and higher lines of prior systemic therapies (HR = 2.229, *P* = 0.040) were independently correlated with worse OS (**Table 6**).

**Table 5:** Univariable Cox regression for OS.

Parameters	HR (95% CI)	*P*-value
Group (DEB-TACE versus cTACE)	0.468 (0.197–1.110)	0.085
Age ≥70 years	1.337 (0.582–3.075)	0.494
Sex—male	1.799 (0.662–4.888)	0.249
Higher ECOG performance score	1.419 (0.593–3.395)	0.431
Child–Pugh stage B	1.311 (0.442–3.889)	0.626
Time to liver metastases <1 year	1.192 (0.487–2.920)	0.701
Bilobar disease	1.483 (0.651–3.380)	0.348
Multifocal disease	2.262 (0.917–5.576)	0.076
Largest diameter of liver metastases ≥5 cm	2.543 (1.091–5.931)	0.031
Primary tumor site		
Reference (rectum)	1.000	
Right colon	1.545 (0.549–4.351)	0.410
Left colon	0.732 (0.194–2.771)	0.646
KRAS mutation	2.344 (0.912–6.022)	0.077
BRAF V600E mutation	2.191 (0.637–7.538)	0.213
Higher lines of prior systemic therapies	1.905 (1.019–3.562)	0.044

OS, overall survival; HR, hazard ratio; CI, confidence interval; DEB-TACE, drug-eluting bead transarterial chemoembolization; cTACE, conventional transarterial chemoembolization; ECOG, Eastern Cooperative Oncology Group; KRAS, Kirsten rat sarcoma viral oncogene homolog; BRAF, v-Raf murine sarcoma viral oncogene homolog B.

**Table 6:** Multivariable Cox regression for OS.

Parameters	HR (95% CI)	*P*-value
Group (DEB-TACE versus cTACE)	0.382 (0.148–0.988)	0.047
Multifocal disease	2.448 (0.925–6.478)	0.071
Largest diameter of liver metastases ≥5 cm	2.453 (1.016–5.921)	0.046
KRAS mutation	1.287 (0.446–3.713)	0.640
Higher lines of prior systemic therapies	2.229 (1.038–4.786)	0.040

OS, overall survival; HR, hazard ratio; CI, confidence interval; DEB-TACE, drug-eluting bead transarterial chemoembolization; cTACE, conventional transarterial chemoembolization; KRAS, Kirsten rat sarcoma viral oncogene homolog.

### 3.6 Comparison of adverse events

The incidence rates of fever (55.0% versus 47.8%, *P* = 0.639), pain (45.0% versus 39.1%, *P* = 0.697), nausea and vomiting (25.0% versus 39.1%, *P* = 0.324), ALT elevation (20.0% versus 26.1%, *P* = 0.637), AST elevation (15.0% versus 21.7%, *P* = 0.571), anemia (10.0% versus 8.7%, *P* = 0.883), leukopenia (10.0% versus 13.0%, *P* = 0.756), and thrombocytopenia (0.0% versus 4.3%, *P* = 0.345) were not different between the DEB-TACE group and the cTACE group (**Table 7**).

**Table 7:** Incidence of adverse events.

Items	cTACE group (*N* = 23)	DEB-TACE group (*N* = 20)	*P*-value
Fever, *n* (%)	11 (47.8)	11 (55.0)	0.639
Pain, *n* (%)	9 (39.1)	9 (45.0)	0.697
Nausea and vomiting, *n* (%)	9 (39.1)	5 (25.0)	0.324
ALT elevation, *n* (%)	6 (26.1)	4 (20.0)	0.637
AST elevation, *n* (%)	5 (21.7)	3 (15.0)	0.571
Anemia, *n* (%)	2 (8.7)	2 (10.0)	0.883
Leukopenia, *n* (%)	3 (13.0)	2 (10.0)	0.756
Thrombocytopenia, *n* (%)	1 (4.3)	0 (0.0)	0.345

cTACE, conventional transarterial chemoembolization; DEB-TACE, drug-eluting bead transarterial chemoembolization; ALT, alanine aminotransferase; AST, aspartate aminotransferase.

### 3.7 Subgroup analysis on the basis of age stratification

Considering the globally recognized threshold of 65 years for elderly definition, this study further stratified patients into those aged ≥65 years (32 cases) and those aged 60–64 years (11 cases) and performed subgroup analysis. As shown in **Table 8**, in the subgroup of patients aged ≥65 years, DEB-TACE achieved a prolonged PFS compared with cTACE (median 11.6 versus 6.1 months, *P* = 0.041), but the CR (*P* = 1.000), ORR (*P* = 0.280), DCR (*P* = 0.144), and OS (*P* = 0.116) were not significantly different between them. Moreover, the incidence of each adverse event did not differ between the DEB-TACE and cTACE treatments. In the subgroup of patients aged 60–64 years, CR (*P* = 0.165), ORR (*P* = 0.137), DCR (*P* = 1.000), PFS (*P* = 0.119), and OS (*P* = 0.176) did not differ significantly between the DEB-TACE and cTACE treatments, and neither was the incidence of each adverse event.

**Table 8:** Subgroup analysis.

Items	cTACE group	DEB-TACE group	*P*-value
Subgroup of patients aged ≥65 years			
* N* (patient number)	16	16	
CR rate, *n* (%)	1 (6.3)	1 (6.3)	1.000
ORR, *n* (%)	5 (31.3)	8 (50.0)	0.280
DCR, *n* (%)	12 (75.0)	15 (93.8)	0.144
PFS, median (95% CI)	6.1 (1.0–11.2)	11.6 (6.7–16.5)	0.041
OS, median (95% CI)	14.1 (10.3–17.9)	18.5 (11.9–25.1)	0.116
Adverse events, *n* (%)			
Fever	9 (56.3)	9 (56.3)	1.000
Pain	6 (37.5)	7 (43.8)	0.719
Nausea and vomiting	8 (50.0)	5 (31.3)	0.280
ALT elevation	5 (31.3)	4 (25.0)	0.694
AST elevation	4 (25.0)	3 (18.8)	0.669
Anemia	2 (12.5)	2 (12.5)	1.000
Leukopenia	3 (18.8)	2 (12.5)	0.626
Thrombocytopenia	1 (6.3)	0 (0.0)	0.310
Subgroup of patients aged 60–64 years			
* N* (patient number)	7	4	
CR rate, *n* (%)	0 (0.0)	1 (25.0)	0.165
ORR, *n* (%)	2 (28.6)	3 (75.0)	0.137
DCR, *n* (%)	7 (100.0)	4 (100.0)	1.000
PFS, median (95% CI)	8.9 (2.7–15.1)	17.1 (NR–NR)	0.119
OS, median (95% CI)	20.7 (1.1–40.3)	NR (NR–NR)	0.176
Adverse events, *n* (%)			
Fever	2 (28.6)	2 (50.0)	0.477
Pain	3 (42.9)	2 (50.0)	0.819
Nausea and vomiting	1 (14.3)	0 (0.0)	0.428
ALT elevation	1 (14.3)	0 (0.0)	0.428
AST elevation	1 (14.3)	0 (0.0)	0.428
Anemia	0 (0.0)	0 (0.0)	1.000
Leukopenia	0 (0.0)	0 (0.0)	1.000
Thrombocytopenia	0 (0.0)	0 (0.0)	1.000

cTACE, conventional transarterial chemoembolization; DEB-TACE, drug-eluting bead transarterial chemoembolization; CR, complete response; ORR, objective response rate; DCR, disease control rate; PFS, progression-free survival; OS, overall survival; CI, confidence interval; ALT, alanine aminotransferase; AST, aspartate aminotransferase.

## 4 Discussion

DEB-TACE and cTACE are performed via transarterial hepatic artery access. cTACE combines chemotherapeutic agents (e.g., irinotecan) with iodized oil and embolic particles to achieve local tumor control via high drug concentrations and embolization. DEB-TACE, by contrast, uses pre-loaded, degradable, or non-degradable microspheres that release chemotherapy slowly, reducing peak plasma concentrations and thereby lowering systemic toxicity and post-embolization syndrome. Both provide effective local control for CRCLM patients refractory to systemic chemotherapy or unsuitable for surgery. Nevertheless, marked patient heterogeneity, such as differing prior treatment lines and extrahepatic metastases, hinders objective comparison between the two techniques. Clinical practice thus depends largely on institutional experience, with guidelines recommending these procedures for liver-confined metastases after systemic therapy failure, underscoring the need for multidisciplinary decision-making (22).

A previous single-arm study reported that DEB-TACE achieved a CR rate of 14.3%–23.8%, an ORR of 78.6%–92.9%, and a DCR of 90.5%–100.0% within 6 months after DEB-TACE treatment in CRCLM patients (15). Another single-arm study reported that DEB-TACE achieved an ORR of 14.3% and a DCR of 54.3% after DEB-TACE treatment in CRCLM patients (16). Only one study reported the comparison between DEB-TACE and cTACE in CRCLM patients with a limited sample size, which revealed that DEB-TACE had a higher tendency in ORR (36.4% versus 18.2%) and DCR (81.8% versus 54.5%) at 3 months compared with cTACE, but it did not reach statistical significance (17). This present study found that the CR rate (10.0% versus 4.3%), ORR (55.0% versus 30.4%), and DCR (95.0% versus 82.6%) showed tendencies to be higher after DEB-TACE compared with cTACE in elderly CRCLM patients, but these did not reach statistical significance. This finding was generally in line with previous studies (15–17). The slight difference in the numerical rates among studies may be due to the different treatment lines of DEB-TACE and the assessed time of response. The explanation for the relatively better tendency of treatment response despite the lack of statistical significance may be that the sample size was not large enough and the statistical power was relatively low.

For the survival benefits, a previous single-arm study reported that DEB-TACE had a median OS of 25.0 (95% CI: 19.3–30.7) months in CRCLM patients (15), another single-arm study reported that DEB-TACE gained a median PFS of 6.3 (95% CI: 2.3–10.3) months and a median OS of 47.4 (95% CI: 21.1–73.7) months in CRCLM patients (16). Interestingly, a double-arm study reported that DEB-TACE achieved a prolonged PFS (median: 12.0 versus 4.0 months) and OS (median: 24.0 versus 14.0 months) over cTACE in CRCLM patients (17). This present study also found that DEB-TACE lengthened the PFS (median: 11.7 versus 6.1 months) and showed a tendency to prolong the OS (median: 21.6 versus 16.3 months) compared with cTACE in elderly CRCLM patients, which were in line with the previous study (17). The comparable survival profile between DEB-TACE and cTACE may be due to the following aspects: first, DEB-TACE uses microspheres that act as drug carriers when blocking the blood vessels, and they can slowly and continuously release chemotherapy drugs (such as irinotecan). This enables the local tumor to maintain an effective drug concentration for a long time, continuously killing tumor cells (22–24). However, cTACE uses conventional iodized oil emulsifier to inject the drugs, which would be washed away by the blood flow, resulting in a relatively rapid decrease in the local drug concentration around the tumor and a shorter contact time with the tumor cells. Second, microspheres used in DEB-TACE are relatively uniform-sized particles that can achieve more precise and thorough embolization of peripheral blood vessels, achieving the effect of “delivery + embolization” in one step. This physical and persistent ischemic hypoxic environment itself has a strong killing effect on tumors (25). However, the embolization effect of conventional iodized oil and gelatin sponge particles in cTACE is often variable due to individual differences and operational techniques. The durability and thoroughness of the embolization are not as good as those of standardized microspheres.

Multivariable Cox regression analyses were performed in this present study, which found that group (DEB-TACE versus cTACE) was independently correlated with a longer PFS and OS, confirming the superiority of DEB-TACE over cTACE in elderly CRCLM patients. Moreover, multivariable Cox regression analyses also found that the largest diameter of liver metastases ≥5 cm and higher lines of prior systemic therapies were independently correlated with a worse PFS and/or OS in elderly CRCLM patients. The explanation for this finding may be as follows: first, a larger liver metastasis (≥5 cm) represents a high tumor burden and a complex blood supply situation, resulting in incomplete treatment; it also restricts the drug penetration and distribution to reduce the treatment efficacy (26). Second, higher lines of prior systemic therapies would lead to a higher risk of multiple drug resistance and tumor invasiveness, thereby resulting in poor treatment outcomes. Moreover, higher lines of prior systemic therapies are often complicated with worse physical condition, high tumor burden, and damaged liver function, reducing the treatment efficacy.

Commonly attended adverse events in DEB-TACE-treated CRCLM patients include fever, pain, nausea and vomiting, ALT elevation or AST elevation, anemia, leukopenia, etc. (15–17). However, seldom study reports the difference in adverse events between DEB-TACE and cTACE in CRCLM patients; while referring to studies in HCC patients, DEB-TACE reveals non-varied adverse events compared to cTACE (27, 28). This present study found that the incidence of adverse events was not different between DEB-TACE and cTACE in the elderly CRCLM patients, which was in accordance with the previous studies focusing on HCC patients (27, 28). These findings suggest that the safety of DEB-TACE is comparable to cTACE in these patients, and provide some evidence for the future application of DEB-TACE.

For potential limitations in this present study: firstly, since the application of TACE in CRCLM patients is less common, and this study focuses on elderly patients which further restricts the sample size; therefore, the sample size of this present study is limited (43 cases), lacking robust statistical power. Secondly, this present study analyzes CRCLM patients with primary CRC resected; thus, the findings are not suitable for those not receiving primary lesion resection. Thirdly, it is recently found that TACE combined with targeted therapies improves the prognosis compared with monotherapy in CRCLM patients (29, 30), implying that the combination of DEB-TACE with target therapies may increase the benefits as well, but this point is not investigated in this present study. Fourthly, this study uses ≥60-year threshold for the definition of elderly, which is a regional administrative definition. This point would limit the direct comparability of these findings with global studies that typically utilize ≥65 or ≥70 years as threshold, and limits the comparability of these findings with the international medical community. To clarify this issue as possible, we add a subgroup analysis in respective patients with ≥65 years and patients with 60–64 years. Fifth, the incidences of adverse events are retrieved from medical records retrospectively, while grading information is not included and analyzed, due to that the relevant records are not documented in detail and do not refer to standard grading criteria; this aspect is a limitation, and further validation of adverse events between DEB-TACE and cTACE is needed in future studies. Sixth, the differences in microsphere sizes and actual irinotecan doses may be confounders affecting the results. Seventh, other confounding factors may also exist such as selection bias among centers and the differing expertise with TACE.

In conclusion, DEB-TACE appears to be a comparable and feasible option compared to cTACE in elderly CRCLM patients. This indicates that DEB-TACE is an optional choice for the treatment of elderly CRCLM patients. However, more future studies are needed for further confirmation.

## Data Availability

The original contributions presented in the study are included in the article/supplementary material. Further inquiries can be directed to the corresponding author.
